# Problems with sex among gay and bisexual men with diagnosed HIV in the United Kingdom

**DOI:** 10.1186/1471-2458-12-916

**Published:** 2012-10-29

**Authors:** Adam Bourne, Ford Hickson, Peter Keogh, David Reid, Peter Weatherburn

**Affiliations:** 1 Sigma Research, Department of Social & Environmental Health Research, London School of Hygiene & Tropical Medicine, London, UK; 2 School of Health and Social Care, University of Greenwich, Mary Seacole Building, Avery Hill Road, London, UK

**Keywords:** HIV, MSM, Positive prevention, Stigma, Sexual dysfunction

## Abstract

**Background:**

A significant research literature exists that details the sexual health and sexual behaviour of gay and bisexual men who have diagnosed HIV. However, much of this research has focussed on HIV transmission risk behaviours among this group, rather than seeking to understand their sexual health and sexual well-being more broadly. There have been growing calls for interventions to support people with diagnosed HIV to achieve health and well-being, including sexual health and well-being. A detailed understanding of the problems people in this group face, and how they might be overcome, is required to facilitate such interventions.

**Methods:**

One thousand two hundred and seventeen gay and bisexual men with diagnosed HIV were recruited by convenience sampling through charitable AIDS service organisations, genitourinary medicine clinics and local authority agencies to complete a survey of their health and social care needs. Respondents were asked to report any problems they had with regards to sex during the 12 months prior to survey completion. They were also asked to describe what support might help them to overcome any problems they experienced.

**Results:**

Overall, 70.5% of the gay and bisexual men with diagnosed HIV completing the survey reported one or more problems with sex within the previous 12 months. Most commonly reported problems include loss of libido (44.0%, n=540), poor self-image or low self confidence (43.9%, n=534), worries about passing HIV to potential sexual partners (37.3%, n=454), and fears of rejection from sexual partners (34.7%, n=422). Responses varied according to age, time since diagnosis, and whether or not the respondent was currently taking anti-retroviral therapy. Qualitative analysis of data relating to what support might help men overcome problems with sex indicate a need for therapeutic support to increase self esteem and confidence, clarity on criminalisation of HIV transmission, the tackling of HIV related stigma and help to achieve a higher quality (as opposed to quantity) of sex.

**Conclusions:**

The findings indicate a need for the maintenance and expansion of services to meet the significant needs of people with diagnosed HIV, especially as these intersect with their ability to negotiate sex that is satisfying.

## Background

There are now approximately 32,000 men who have sex with men (MSM) in the UK living with diagnosed HIV. [1] While the sexual behaviour of MSM living with diagnosed HIV has been the focus of research [2-4] and HIV sexual health promotion practice for many years, this has typically focussed on the HIV related risks they pose to their sexual partners (although for notable exceptions see Asboe et al. [5] and Harding et al. [6]). This notion of ‘Positive Prevention’ largely relates to encouraging and empowering people living with HIV to take precautionary measures to ensure they do not pass on HIV to their sexual partners. Such an approach has been criticised for placing the burden of responsibility for preventing infections on the shoulders of HIV-positive people.

In recent years there has been increasing recognition that rewarding sex, good mental health and positive well-being for the person living with diagnosed HIV should be integral components of Positive Prevention. [7] Indeed, the turn of the decade has witnessed a reshaping of terminology that now promotes ‘Positive Health, Dignity and Prevention’. [8] The primary goal of this approach is to support people living with HIV/AIDS to achieve health and well-being, including sexual health and well-being. In England, the CHAPS partnership of HIV health promotion organisations has recently published its revised strategic planning framework which, more than ever before, establishes the promotion of the best sexual health for MSM living with HIV as a goal of its activities. [9]

A growing body of evidence suggests significant sexual ill-health and social isolation for individuals living with HIV. Previous authors [10] have reported experiences of stigma, rejection and significant mental ill-health amongst the group of gay identifying men living with HIV in England. Others have described sexual difficulties and an association with depression amongst HIV-positive gay men in Australia [11]. Sexually transmitted infection (STI) incidence and diagnosis data from the Health Protection Agency indicate significantly higher rates of syphilis and lymphogranuloma venereum (LGV) transmission among MSM with diagnosed HIV. [12,13] A rising incidence of Hepatitis C among MSM with diagnosed HIV has been observed in several countries [14,15].

Previous survey research among people with diagnosed HIV in the UK found 51% of all respondents to have experienced a problem or problems with sex in the preceding 12 months [16]. The most commonly described problems were little or no sex, erectile dysfunction, HIV transmission anxiety and poor self-image. In the years since that study was published there has been a number of clinical, social and legal developments in the UK that have the potential to impact on the sexual behaviour of people with diagnosed HIV, including a trend toward criminal prosecutions for HIV transmissions [17] and continuing advances in anti-retroviral therapies and clinical outcomes for individuals on treatment. [18,19] Given this changing social, legal, and clinical environment, there is a need for more data relating to the current sexual health needs of this group. This paper explores: (1) the problems that gay men with diagnosed HIV experience with regards to sex and how are these associated and; (2) the ways in which these sexual problems might be alleviated.

## Methods

Data from this paper are derived from *What do you need? 20072008,* a broad ranging needs assessment of people living with diagnosed HIV (PLWHIV) in the UK whose detailed methods have been published elsewhere. [20] The following outlines its key features.

### Sample recruitment

The survey used a self-completion questionnaire with attached reply-paid envelope. Distribution occurred between 5^th^ June 2007 and 5^th^ January 2008. As there is no national sampling frame available, recruitment was by convenience. Booklet versions of the questionnaire were distributed by 107 different agencies across the UK, including charitable AIDS service organizations, genitourinary medicine or HIV out-patients clinics, and local authority agencies. Overall, 17815 booklets were requested by and sent out to these agencies. The HIV charity *NAM* also sent the booklet questionnaire by post to 4950 subscribers to their HIV treatments newsletter (now called *HIV Treatment Update*). An identical questionnaire was also available to complete online (hosted by http://www.demographix.com) in both English and French. Its availability was promoted via the websites of eleven collaborating HIV health promotion agencies.

### Measures

Twenty areas of need were chosen for inclusion in the survey. Seventeen of these were derived from an earlier version of the survey [16] while others (‘Family relationships’, ‘Looking after children’, and ‘Immigration’) were derived from discussions with HIV health and social care professionals in the field. This paper focuses only on responses to questions regarding sex.

Respondents were asked: *In the past 12 months have you had any problems with your sex life?* Those who indicated ‘yes’ were asked to clarify what those problems were from a list of 10 problems derived from open-ended responses to the same question in a previous survey. [16] Respondents could also describe ‘Other’ problems. Those who had experienced one or more problems were asked whether they had sought help or support in relation to their problems. They were then asked: *Compared to a year, ago have these problems got better, worse or gone away?* Those who indicated their problems had got better, worse or had experienced no change (but not those who said their problem had gone away) were asked: *With more help or support do you think you could reduce or overcome these problems?* Those indicating ‘yes’ or ‘don’t know’ were asked *Realistically, what help do you think would make a difference to your sex life?* with a free-response box.

### Analysis

Descriptive quantitative analysis was undertaken with SPSS PC version 18.0. Chi-square and Student-t tests were used to establish sample differences according to age, time living with diagnosed HIV, and region or country of UK residence. The ten specific problems were correlated with each other to give a matrix which was then factor analysed using principal components analysis with Varimax rotation. Factors with loadings of 0.7 and above were considered and those items so loaded were used to interpret the factors. Qualitative responses to the question of what help and support for unresolved problems might look like were subjected to a thematic content analysis. The data were read and reread and initial codes (interesting features) were documented. These were then collated into relevant themes and all examples of each potential theme were recorded. After reviewing the themes to ensure they accurately represented the source material from which they came, they were labelled and described in narrative form.

### Ethics

Information provided at the beginning of the survey outlined the motivations for, and protection of, the data. Consent was assumed by any subsequent completion of the survey. The study received approval from Oxford A Multi-centre Research Ethics Committee with confirmation that the research fell within the Department of Health's (November 2000) Supplementary Operational Guidelines for NHS Research Ethics Committees on Multi-Centre Research in the NHS.

## Results

### Response and sample characteristics

Overall, we received 1929 questionnaires, including 1113 booklets and 816 online responses. Of these, 152 responses (8.6% of all) were excluded from further analysis because they did not live within the UK (n=33), and/or did not confirm they had diagnosed HIV (n=103) and / or had previously taken part in the study (n=36). This left a final sample of 1777 unique individuals with diagnosed HIV resident in the UK. This paper reports responses from the 1217 male respondents who identified as gay or bisexual, or who specified a term for their sexuality that implied same-sex behavior.

As a group, men who completed the booklet version of the survey had been diagnosed with HIV for longer compared to men who completed the online version (F=93.1, df =1, p<0.001). Those who completed the booklet version were significantly more likely to be on HIV anti-retroviral therapy than those who completed the survey online (83.9% versus 62.1%; χ^2^ = 72.7, df = 1, p<0.01). Men who had completed the survey online were as a group significantly younger than men who completed the booked version (F=126.94, df = 1 , p<0.001) There were no significant differences between booklet and online recruited men according to ethnicity, level of educational attainment, region of residence or sexual identity.

The difference between our sample and all men living in the UK with diagnosed homosexually acquired HIV was estimated by comparing sample demographics with the National Survey of Prevalent HIV Infections Diagnosed (SOPHID), conducted annually by the Health Protection Agency’s Centre for Infections and Health Protection Scotland. [21] SOPHID 2007 data was used. Our sample over-represents men in Wales and Northern Ireland and under-represents men in Scotland. Across England, our sample over-represents men in the North West and West Midlands regions and under-represents those in East of England and East Midlands. The sample also over-represents men of white British ethnicity and under-represents men from all minority ethnic groups (Table 1).

**Table 1:** Demographic profile of gay and bisexual men in the sample (N=1217)

**Country or region of residence**	**% (n)**	*SOPHID%	**Highest educational level**	**% (n)**
All England	91.5 (1108)	91.6	None	1.2 (14)
*East of England*	*3.5 (43)*	*2.8*	Primary/Elementary	<1 (5)
*East Midlands*	*2.7 (33)*	*4.0*	Secondary/High School	26.9 (327)
*London*	*44.3 (538)*	*47.1*	University/College	71.2 (862)
*North East*	*2.3 (28)*	*1.9*		
*North West*	*11.8 (142)*	*10.7*		
*South Central*	*3.8 (46)*	*3.4*		
*South East Coast*	*7.8 (96)*	*8.2*	**Age**	
*South West*	*3.8 (47)*	*4.8*	Mean	42.9
*West Midlands*	*7.1 (87)*	*5.0*	Median	42.0
*Yorkshire & Humber*	*4.1 (48)*	*3.6*	Range	17-76
All Wales	4.3 (52)	2.3		
All Scotland	2.8 (34)	4.7		
All Northern Ireland	1.2 (15)	0.8		
**Ethnicity**	**% (n)**	*SOPHID%	**Time since HIV diagnosis**	**% (n)**
White	94.6 (1146)	87.2	<2 years	19.1 (229)
Black African	0.4 (5)	1.3	2-<5 years	21.3 (256)
Black Caribbean	0.7 (9)	2.1	5-<10 years	21.5 (259)
Black other	0.2 (2)	1.1	>10 years	38.1 (458)
Indian/Pakistani/Bangladeshi	0.8 (10)	1.1		
Other Asian/Oriental	0.8 (10)	1.5		
Other and mixed	2.4 (29)	4.9		
**Receiving antiretroviral therapy**	**% (n)**	*SOPHID%	**Sexuality**	**(% (n)**
Yes	73.9 (884)	70.3	Gay	94.7 (1152)
No	26.1 (313)	28.4	Bisexual	4.9 (60)
			Other	<1 (5)

* 2007 SOPHID data for homosexually acquired HIV [21].

### Prevalence of problems with sex

Overall, 70.5% (N=1199, missing 18) of the MSM with diagnosed HIV reported one or more problems with sex within the previous 12 months. There was no significant difference in whether or not a problem was reported by age, time since diagnosis with HIV, or area of residence, although specific problems did show difference (see below). The problems experienced are shown in Table 2.

**Table 2:** Percentage of respondents reporting each of ten specified problems with sex

**Problems with sex among all respondents (N=1217)**	**N**	**%**
Loss of libido	540	44.0%
Poor self-image/low self-confidence	534	43.9%
Too little or no sex	501	41.2%
Worries about passing HIV to partners	454	37.3%
Fear of rejection from potential partners	422	34.7%
Worries about disclosing HIV to partners	387	31.8%
Problems with penis or anus	346	28.4%
Worries about prosecution if HIV is spread	295	24.2%
Treatment side effects	216	17.7%
Too much sex	49	4.0%
Other problems	52	4.3%

Among *Other problems*, respondents most commonly described sexual pleasure being undermined by illness, pain and/or treatment side-effects. Fatigue, problems with bowel or prostate, and skin disorders were also mentioned.

The problems experienced varied by age but not all in the same direction. Men who had a problem with disclosing their HIV status to sexual partners were as a group significantly younger than men who did not have this problem (F=17.6, df = 1,874, p<0.001). This was also true in relation to worries about passing HIV to a sexual partner (F=24.12, df = 1,874, p<0.001) and worries about prosecution if HIV is transmitted (F=9.96, df = 1,874, p<0.01). Men who had a problem with poor self image or low self-confidence were, on the other hand, older (F=18.63, df = 1,874, p<0.001).

Several problems were more common among men not currently on anti-retroviral therapy. Compared to men on therapy, those not on therapy were more likely to have been worried about disclosing their HIV status to a sexual partner (54.7% versus 42.2%; χ^2^ = 10.12, df = 1, p<0.01), to fear rejection from a sexual partner (57.5% versus 46.6%; χ^2^ = 7.59, p<0.05) and to worry about transmitting HIV to a sexual partner (66.4% versus 48.8%; χ^2^ = 19.99, df = 1, p<0.001).

Men diagnosed within the preceding 5 years were more likely to be worried about disclosing their HIV status (55.0% versus 39.0%; χ^2^ = 21.216, df = 1, p<0.001) and about transmitting HIV to a sexual partner (64.6% versus 45.9%; χ^2^ = 28.75, df = 1, p<0.001) than men who had been diagnosed for over 5 years.

### Groupings of problems with sex

In factor analysis, the first four components extracted accounted for 62.6% of the variance in the matrix, and were interpretable. Their loadings for the ten items are shown in Table 3. No other factor had a loading of 0.7 or above on any of the ten items.

**Table 3:** Factor loadings for the first 4 principal components

**Problem**	**Factor**			
	**1**	**2**	**3**	**4**
Too little or no sex	-.008	.120	**.824**	.297
Problems with penis or anus	.167	.579	-.354	-.194
Loss of libido	.073	**.730**	-.042	-.199
Too much sex	.193	-.226	-.501	**.715**
Worries about disclosing HIV to sex partners	**.799**	-.187	.013	-.148
Worries about being prosecuted if HIV is passed on	**.746**	-.161	-.024	-.179
Fear of rejection from potential sexual partners	**.805**	-.005	.102	-.018
Poor self image / low self confidence	.513	.311	.238	.307
Worries about passing HIV on to sexual partners	.696	-.165	-.015	-.096
Treatment side effects	.223	.554	-.098	.391

The first factor (accounting for 27.1% of the variance) loaded at almost 0.7 and above on three items related to managing disclosure to sexual partners: worries about sexual rejection, worries about disclosure and worries about being prosecuted for passing on HIV following non-disclosure. Worries about passing on HIV also loaded heavily on this factor. The following three factors each loaded at 0.7 or over on one item only – loss of libido, too little or no sex, and too much sex.

### Potential for resolution of sexual problems

Men who had experienced a problem in relation to sex in the last year were asked whether their problems had been solved or gone away, gotten better, gotten worse or stayed the same. Only 1.2% (n=10) felt that the problem had been solved or gone away, 14.1% (n=118) said things had gotten better and for 34.4% (n=289) things had gotten worse. 50.3% (n=422) reported no change. Of those who indicated their problems had gotten better, worse or had not changed, 35.7% (n=287) felt that further help or support could help them to reduce or overcome these problems, and a further 40.1% (n=322) were unsure whether it could.

### Suggestions for support

Four key themes emerged from the suggestions men made for what this support might realistically look like (n=393). These were: therapeutic support to increase self-esteem and confidence; tackling HIV-related stigma; clarity on criminalisation of HIV transmission; and help to achieve better quality of sexual life. Short descriptions of themes with indicative quotes from respondents now follow.

#### Therapeutic support to increase self-esteem and confidence

Over a third of responses related to a desire for therapeutic support, either group-based or one-to-one, to help address issues of low self-esteem or low self-confidence, which were having a negative impact on their ability to have sex they are happy with. Typical responses were “*Feeling better about myself and not seeing myself as a dirty, infected bastard would help things*” and *“Continued counselling support to build confidence.”*

#### Tackling HIV related stigma

Around a quarter of respondents said that tackling HIV-related stigma, or broadly educating other gay men and the general public about HIV (including how it is transmitted and what the prognosis now is) would help to improve their sexual interactions and reduce the likelihood of rejection following disclosure. A typical response was “*Finding that the level of public understanding was sufficiently improved that I felt more confident about disclosure to prospective partners without risking ostracisation or public humiliation*.”

#### Clarity on criminalisation of HIV transmission

Around a sixth of responses directly related to concerns about the criminalisation of HIV transmission and a desire for clearer guidance for men, their sexual partners, and health professionals about how and why such prosecutions operate. Most were critical of the use of the criminal law and the consequences for risk negotiation. A typical response was “*Concern about transmission and criminalisation makes me anxious and depressed…a more reasoned discussion about criminalisation would help. Currently, all the pressure and responsibility is on ME…what happened to each person is responsible for their own sexual health?*”

#### Help to achieve higher quality of sexual life

Around a quarter of respondents expressed a need for help achieving good quality sex, rather than simply a high frequency of sexual contact. They also wanted help to overcome issues with erectile dysfunction as well as a loss of libido, which were having a detrimental impact on their sex lives. Typical responses were “*I want more than just sex that is easy to get but these days I want sex WITHIN a relationship not recreational sex*” and *“Safe treatment for erectile problems.”*

## Discussion

Our survey has identified key areas for sexual health promotion among MSM with diagnosed HIV, although some shortcomings of the survey should be noted. By using multiple recruitment methods, we sought to reduce the biases of opportunistic recruitment. While a relatively small number of those whom came into contact with the survey actually completed it, the final sample represents 5% of MSM with diagnosed HIV seen for care in 2007. [21] The sample will not include people who do not have any contact with services, unless given a questionnaire by friends. It will also be skewed away from people who are not literate in English, worried about confidentiality or wary of social research. The sample likely over-represents men who are more comfortable both with their homosexuality and with their HIV status. In addition, the sample under-represents ethnic minority MSM with HIV, whose sexual health needs may have additional dimensions.

Our findings highlight significant sexual health need among gay and bisexual men with diagnosed HIV. In terms of the groupings of problems HIV positive MSM experience (and subsequently the tailoring of interventions to groups of men with similar problems), our analysis suggests a large and singularly coherent problem of managing status disclosure to potential sexual partners, with its attendant risks of rejection (or worse) if disclosure occurs, and potential prosecution if disclosure does not occur (but HIV transmission does). Previous qualitative research also reports sometimes harrowing cases of rejection by sexual partners following HIV status disclosure [22], which itself has an impact on self-confidence. A survey of 7461 gay and bisexual men with diagnosed HIV found that if faced with HIV status disclosure by a potential sexual partner, 51.9% of HIV negative or untested men would not wish to have sex at all. [23] However, similar research in 2006 found that 74.3% of gay and bisexual men expect potential sexual partners with diagnosed HIV to disclose their status prior to sex. [24] Uncertainty about their obligations to disclose under the criminal law, and a justifiable fear of rejection and its possible consequences, make it harder for men with diagnosed HIV to talk openly about their status and negotiate risk management strategies that are appropriate for their situation.

Two separate and distinct sexual problem areas related to *not* having sex emerged from the analysis, these concerned loss of libido and not wanting sex (Factor 2), and wanting sex but not having (sufficient) opportunities to engage in it (Factor 3). Similar survey research with people with diagnosed HIV in Australia also reported that 57.6% of respondents felt having HIV had negatively impacted on their libido. [25]

A significant number of men reported problems related to low self-esteem or low self-confidence. Such issues were represented in several other needs explored in the *What do you need?* survey. [20] A disproportionate burden of mental ill-health among sexual minorities more broadly, and people with diagnosed HIV specifically, has been well-documented [26-28], but the current data highlight the significant negative impact this can have on sexual happiness and sexual well-being.

Collectively, these data emphasise a need for the maintenance and expansion of mental health services to meet the significant needs of people with diagnosed HIV, especially as these intersect with their ability to negotiate sex that is satisfying. Organisations working to improve the sexual health and well-being of men with diagnosed HIV always need to remain proactive in their approach – highlighting the problems that many positive men face as a means of normalising them, and advertising the help they can provide to try and overcome them. People designing programs or policy initiatives should note that problems are not distributed evenly among men with diagnosed HIV. Rather problems with disclosure, and concerns relating to criminal prosecution if HIV is transmitted, seem particularly salient for younger men, while older men are more likely to have problems with poor self-image or low self-confidence.

## Conclusions

Data collected as part of this study indicate that there is considerable unmet sexual health need among gay and bisexual men with diagnosed HIV, particularly as it relates to loss of libido, managing information about ones HIV status, and in terms of low self-esteem or self-confidence. Like all homosexually active men, those with diagnosed HIV have a right to the best sex with the least harm. Meeting their sexual needs, helping them to overcome issues of self-esteem or self-confidence will help them have more rewarding sex lives, while empowering them to negotiate sex more effectively and addressing HIV-related stigma may also facilitate an environment in which onward transmission of HIV is less likely to occur.

## Competing interests

The authors declare that they have no competing interests.

## Authors' contributions

AB conducted principal data analysis and wrote the first draft of the manuscript. FH contributed additional statistical analysis and helped to conceptualise the focus of enquiry. PK, DR and PW participated in the design and execution of the study and helped to draft the manuscript. All authors read and approved the final manuscript.

## Pre-publication history

The pre-publication history for this paper can be accessed here:

http://www.biomedcentral.com/1471-2458/12/916/prepub
